# PRESIDENTIAL SYMPOSIUM: EDUCATION NETWORKS: STRENGTHENING GERONTOLOGY AND GERIATRICS THROUGH CONNECTIVITY

**DOI:** 10.1093/geroni/igz038.1574

**Published:** 2019-11-08

**Authors:** Judith L Howe, Kathryn Hyer

**Affiliations:** 1 James J Peters VA Medical Center, GRECC, Bronx, New York, United States; 2 University of South Florida, Tampa, Florida, United States

## Abstract

The AGHE Presidential Symposium, related to the theme of the annual scientific meeting, underscores the importance of networks, collaborations and partnerships in advancing education in gerontology and geriatrics. AGHE has been at the forefront of many innovative programs since it was founded in 1974, contributing to the growth of the field and the recognition of education as one pillar of the field of gerontology and geriatrics, along with research, policy and practice. This symposium highlights three ongoing initiatives that promote connections and collaborations. The first paper discusses the Age-Friendly University (AFU) network which is made of institutions around the globe who have committed themselves to becoming more age-friendly in their programs and policies. AGHE endorses the AFU principles and invites its members and affiliates to call upon their institutions become part of this pioneering initiative. The AFU initiative is one of several international activities that AGHE, global leaders in education on aging, has engaged in. The second paper describes international networking activities such as collaborations with international organizations including the World Health Organization and connecting international and US students. In the third paper, initiatives to connect disciplines and professions through competency-based education and curricula are discussed. For instance, the Gerontology Competencies for Undergraduate and Graduate Education and the Program of Merit promote competency-based gerontology education across disciplines and professions.

